# Association between a large change between the minimum and maximum monthly values of solar insolation and a history of suicide attempts in bipolar I disorder

**DOI:** 10.1186/s40345-024-00364-5

**Published:** 2024-12-23

**Authors:** Philipp Ritter, Tasha Glenn, Eric D. Achtyes, Martin Alda, Esen Agaoglu, Kürsat Altınbaş, Ole A. Andreassen, Elias Angelopoulos, Raffaella Ardau, Memduha Aydin, Yavuz Ayhan, Christopher Baethge, Rita Bauer, Bernhard T. Baune, Ceylan Balaban, Claudia Becerra-Palars, Aniruddh P. Behere, Prakash B. Behere, Habte Belete, Tilahun Belete, Gabriel Okawa Belizario, Frank Bellivier, Robert H. Belmaker, Francesco Benedetti, Michael Berk, Yuly Bersudsky, Şule Bicakci, Harriet Birabwa-Oketcho, Thomas D. Bjella, Conan Brady, Jorge Cabrera, Marco Cappucciati, Angela Marianne Paredes Castro, Wei-Ling Chen, Eric Y. W. Cheung, Silvia Chiesa, Margarita Chanopoulou, Marie Crowe, Alessandro Cuomo, Sara Dallaspezia, Pratikkumar Desai, Seetal Dodd, Bruno Etain, Andrea Fagiolini, Frederike T. Fellendorf, Ewa Ferensztajn-Rochowiak, Jess G. Fiedorowicz, Kostas N. Fountoulakis, Mark A. Frye, Pierre A. Geoffroy, Michael J. Gitlin, Ana Gonzalez-Pinto, John F. Gottlieb, Paul Grof, Bartholomeus C. M. Haarman, Hirohiko Harima, Mathias Hasse-Sousa, Chantal Henry, Lone Hoffding, Josselin Houenou, Massimiliano Imbesi, Erkki T. Isometsä, Maja Ivkovic, Sven Janno, Simon Johnsen, Flávio Kapczinski, Grigorios N. Karakatsoulis, Mathias Kardell, Lars Vedel Kessing, Seong Jae Kim, Barbara König, Timur L. Kot, Michael Koval, Mauricio Kunz, Beny Lafer, Mikael Landén, Erik R. Larsen, Rasmus W. Licht, Vera M. Ludwig, Carlos Lopez-Jaramillo, Alan MacKenzie, Helle Østergaard Madsen, Simone Alberte Kongstad A. Madsen, Jayant Mahadevan, Agustine Mahardika, Mirko Manchia, Wendy Marsh, Monica Martinez-Cengotitabengoa, Julia Martini, Klaus Martiny, Yuki Mashima, Declan M. McLoughlin, Alie N. R. Meesters, Ybe Meesters, Ingrid Melle, Fátima Meza-Urzúa, Elisabeth Michaelis, Pavol Mikolas, Yee Ming Mok, Scott Monteith, Muthukumaran Moorthy, Gunnar Morken, Enrica Mosca, Anton A. Mozzhegorov, Rodrigo Munoz, Starlin V. Mythri, Fethi Nacef, Ravi K. Nadella, Takako Nakanotani, René Ernst Nielsen, Claire O’Donovan, Adel Omrani, Yamima Osher, Uta Ouali, Maja Pantovic-Stefanovic, Pornjira Pariwatcharakul, Joanne Petite, Johannes Petzold, Andrea Pfennig, Maximilian Pilhatsch, Yolanda Pica Ruiz, Marco Pinna, Maurizio Pompili, Richard Porter, Danilo Quiroz, Francisco Diego Rabelo-da-Ponte, Raj Ramesar, Natalie Rasgon, Woraphat Ratta-apha, Maria Redahan, M. S. Reddy, Andreas Reif, Eva Z. Reininghaus, Jenny Gringer Richards, Janusz K. Rybakowski, Leela Sathyaputri, Angela M. Scippa, Christian Simhandl, Daniel Smith, José Smith, Paul W. Stackhouse, Dan J. Stein, Kellen Stilwell, Sergio Strejilevich, Kuan-Pin Su, Mythily Subramaniam, Ahmad Hatim Sulaiman, Kirsi Suominen, Andi J. Tanra, Yoshitaka Tatebayashi, Wen Lin Teh, Leonardo Tondo, Carla Torrent, Daniel Tuinstra, Takahito Uchida, Arne E. Vaaler, Eduard Vieta, Biju Viswanath, Carlo Volf, Kai-Jie Yang, Maria Yoldi-Negrete, Oguz Kaan Yalcinkaya, Allan H. Young, Yosra Zgueb, Peter C. Whybrow, Michael Bauer

**Affiliations:** 1 Department of Psychiatry and Psychotherapy. Faculty of Medicine and University Hospital Carl Gustav Carus, TUD Dresden University of Technology, 01307 Dresden, Germany; 2 ChronoRecord Association, Fullerton, CA USA; 3 Department of Psychiatry, Western Michigan University Homer Stryker M.D. School of Medicine, Kalamazoo, MI USA; 4 Department of Psychiatry, Dalhousie University, Halifax, NS Canada; 5 Department of Psychiatry, Faculty of Medicine, Hacettepe University, Ankara, Turkey; 6 Department of Psychiatry, Atlas University, Istanbul, Turkey; 7 Division of Mental Health and Addiction, Oslo University Hospital & Institute of Clinical Medicine, University of Oslo, Oslo, Norway; 8 Department of Psychiatry, Medical School, Eginition Hospital, National and Capodistrian University of Athens, Athens, Greece; 9 Section of Neurosciences and Clinical Pharmacology, Department of Biomedical Sciences, University of Cagliari, Sardinia, Italy; 10 Department of Psychiatry, Faculty of Medicine, Selcuk University, Konya, Turkey; 11 Department of Psychiatry and Psychotherapy, Faculty of Medicine, University of Cologne, Cologne, Germany; 12 Department of Psychiatry, University of Münster, Münster, Germany; 13 Department of Psychiatry, Melbourne Medical School, The University of Melbourne, Melbourne, Australia; 14 The Florey Institute of Neuroscience and Mental Health, The University of Melbourne, Parkville, VIC Australia; 15 Department of Psychiatry, Psychosomatic Medicine and Psychotherapy, University Hospital Frankfurt, Johann Wolfgang Goethe- Universität Frankfurt Am Main, Frankfurt Am Main, Germany; 16 National Institute of Psychiatry ‘“Ramón de La Fuente Muñiz”, Mexico City, Mexico; 17 Department of Pediatrics and Human Development, Michigan State University, Grand Rapids, MI USA; 18 Department of Psychiatry, Jawaharlal Nehru Medical College, Datta Meghe Institute of Medical Sciences (Deemed University), Wardha, India; 19 Department of Psychiatry, College of Medicine and Health Sciences, Bahir Dar University, Bahir Dar, Ethiopia; 20 Department of Psychiatry, Bipolar Disorder Research Program, University of São Paulo Medical School, São Paulo, Brazil; 21 Département de Psychiatrie Et de Médecine Addictologique, Assistance Publique – Hôpitaux de Paris, INSERM, UMR-S1144, Université Paris-Cité, FondaMental Foundation, Paris, France; 22 Ben Gurion University of the Negev, Beer Sheva, Israel; 23 University Vita-Salute San Raffaele, Milan, Italy; 24 Irccs Ospedale San Raffaele, Milan, Italy; 25 IMPACT – the Institute for Mental and Physical Health and Clinical Translation, School of Medicine, Barwon Health, Deakin University, Geelong, Australia; 26 Orygen, The National Centre of Excellence in Youth Mental Health, Centre for Youth Mental Health, Florey Institute for Neuroscience and Mental Health and the Department of Psychiatry, The University of Melbourne, Melbourne, Australia; 27 Department of Psychiatry, Faculty of Health Sciences, Beer Sheva Mental Health Center, Ben Gurion University of the Negev, Beer Sheva, Israel; 28 Department of Psychiatry, Faculty of Medicine, Baskent University, Ankara, Turkey; 29 Butabika Hospital, Kampala, Uganda; 30 Department of Psychiatry, Trinity College Dublin, St Patrick’s University Hospital, Dublin, Ireland; 31 Mood Disorders Clinic, Dr. Jose Horwitz Psychiatric Institute, Santiago de Chile, Chile; 32 Department of Mental Health and Substance Abuse, Piacenza, Italy; 33 Department of Psychiatry, Chiayi Branch, Taichung Veterans General Hospital, Chiayi, Taiwan; 34 Private Practice, Central, Hong Kong; 35 3rd Department of Psychiatry, School of Medicine, Faculty of Health Sciences, Aristotle University of Thessaloniki, Thessaloniki, Greece; 36 Department of Psychological Medicine, University of Otago, Christchurch, New Zealand; 37 Department of Molecular Medicine, University of Siena School of Medicine, Siena, Italy; 38 Pine Rest Christian Mental Health Services, Grand Rapids, MI USA; 39 Department of Psychiatry, University of Melbourne, Parkville, VIC Australia; 40 Division of Psychiatry and Psychotherapeutic Medicine, Medical University Graz, Graz, Austria; 41 Department of Adult Psychiatry, Poznan University of Medical Sciences, Poznan, Poland; 42 The Ottawa Hospital and University of Ottawa, Ottawa, ON Canada; 43 Department of Psychiatry & Psychology, Mayo Clinic Depression Center, Mayo Clinic, Rochester, MN USA; 44 Département de Psychiatrie et d’addictologie, DMU Neurosciences, AP-HP, GHU Paris Nord, Hopital Bichat - Claude Bernard, 75018 Paris, France; 45 Centre ChronoS, GHU Paris - Psychiatry & Neurosciences, 1 Rue Cabanis, 75014 Paris, France; 46 NeuroDiderot, Université de Paris, 75019 Paris, Inserm France; 47 Department of Psychiatry, GHU Paris Psychiatrie & Neurosciences, 75014 Paris, France; 48 Université de Paris, 75006 Paris, France; 49 Department of Psychiatry and Biobehavioral Sciences, Semel Institute for Neuroscience and Human Behavior, University of California Los Angeles (UCLA), Los Angeles, CA USA; 50 BIOARABA. Department of Psychiatry, University Hospital of Alava, University of the Basque Country, CIBERSAM, Vitoria, Spain; 51 Department of Psychiatry, Feinberg School of Medicine, Northwestern University, Chicago, IL USA; 52 Mood Disorders Center of Ottawa and the Department of Psychiatry, University of Toronto, Toronto, Canada; 53 Department of Psychiatry, University Medical Center Groningen, University of Groningen, Groningen, Netherlands; 54 Department of Psychiatry, Tokyo Metropolitan Matsuzawa Hospital, Setagaya, Tokyo, Japan; 55 Department of Psychiatry, Universidade Federal do Rio Grande do Sul, Porto Alegre, Brazil; 56 Programa de Pós-Graduação Em Psicologia, Departamento de Psicologia do Desenvolvimento e da Personalidade, Instituto de Psicologia, Universidade Federal do Rio Grande do Sul, Porto Alegre, Brazil; 57 Department of Clinical Research, University of Southern Denmark, Odense, Denmark; 58 Université Paris Est Créteil, INSERM, IMRB, Translational Neuropsychiatry, APHP, Mondor Univ Hospitals, Fondation FondaMental, 94010 Créteil, France; 59 Université Paris Saclay, CEA, Neurospin, 91191 Gif-Sur-Yvette, France; 60 Department of Psychiatry, University of Helsinki and Helsinki University Hospital, Helsinki, Finland; 61 National Institute for Health and Welfare, Helsinki, Finland; 62 Clinic for Psychiatry, University Clinical Center of Serbia, Belgrade, Serbia; 63 Department of Psychiatry, University of Tartu, Tartu, Estonia; 64 Unit for Psychiatric Research, Aalborg University Hospital, Aalborg, Denmark; 65 Department of Psychiatry and Neurochemistry, Institute of Neuroscience and Physiology, The Sahlgrenska Academy, University of Gothenburg, Gothenburg, Sweden; 66 Copenhagen Affective Disorder Research Center (CADIC), Psychiatric Center Copenhagen, Rigshospitalet, Copenhagen, Denmark; 67 Department of Psychiatry, Chosun University School of Medicine, Gwangju, Republic of Korea; 68 BIPOLAR Zentrum Wiener Neustadt, Wiener Neustadt, Austria; 69 Khanty-Mansiysk Clinical Psychoneurological Hospital, Khanty-Mansiysk, Russia; 70 Department of Neuroscience, Michigan State University, East Lansing, MI USA; 71 Department of Medical Epidemiology and Biostatistics, Karolinska Institutet, Stockholm, Sweden; 72 Mental Health Department Odense, University Clinic and Department of Regional Health Research, University of Southern Denmark, Esbjerg, Denmark; 73 Psychiatry – Aalborg University Hospital, Aalborg, Denmark; 74 Department of Clinical Medicine, Aalborg University, Aalborg, Denmark; 75 Mood Disorders Program, Department of Psychiatry, Faculty of Medicine, Hospital Universitario San Vicente Fundación, Research Group in Psychiatry, Universidad de Antioquia, Medellín, Colombia; 76 Forensic Psychiatry, University of Glasgow, NHS Greater Glasgow and Clyde, Glasgow, UK; 77 Copenhagen University Hospitals, Psychiatric Centre Copenhagen, Copenhagen, Denmark; 78 Department of Psychiatry, National Institute of Mental Health and Neuro Sciences (NIMHANS), Bengaluru, India; 79 Department of Psychiatry, Faculty of Medicine, Mataram University, Mataram, Indonesia; 80 Department of Pharmacology, Dalhousie University, Halifax, NS Canada; 81 Section of Psychiatry, Department of Medical Science and Public Health, University of Cagliari, Cagliari, Italy; 82 Unit of Clinical Psychiatry, University Hospital Agency of Cagliari, Cagliari, Italy; 83 Department of Psychiatry, University of Massachusetts Medical School, Worcester, MA USA; 84 Osakidetza, Basque Health Service, BioAraba Health Research Institute, University of the Basque Country, Bilbao, Spain; 85 The Psychology Clinic of East Anglia, Norwich, UK; 86 Department of Neuropsychiatry, Keio University School of Medicine, Tokyo, Japan; 87 Department of Psychiatry & Trinity College Institute of Neuroscience, Trinity College Dublin, St Patrick’s University Hospital, Dublin, Ireland; 88 Department of Child and Adolescent Psychiatry Und Psychotherapy, SHG Klinikum, Idar-Oberstein, Germany; 89 Department of Mood and Anxiety Disorders, Institute of Mental Health, Singapore City, Singapore; 90 Michigan State University College of Human Medicine, Traverse City Campus, Traverse City, MI USA; 91 Department of Mental Health, St Olav University Hospital, Trondheim, Norway; 92 Faculty of Medicine and Health Sciences, Norwegian University of Science and Technology – NTNU, Trondheim, Norway; 93 Soviet Psychoneurological Hospital, Urai, Russia; 94 Department of Psychiatry, University of California San Diego, San Diego, CA USA; 95 Makunda Christian Leprosy and General Hospital, Bazaricherra, Assam 788727 India; 96 Razi Hospital, Faculty of Medicine, University of Tunis-El Manar, Tunis, Tunisia; 97 Affective Disorders Research Project, Tokyo Metropolitan Institute of Medical Science, Setagaya, Tokyo, Japan; 98 Tunisian Bipolar Forum, Érable Médical Cabinet 324, Lac 2, Tunis, Tunisia; 99 Department of Psychiatry, Faculty of Medicine Siriraj Hospital, Mahidol University, Bangkok, Thailand; 100 Hospital “Ángeles del Pedregal”, Mexico City, Mexico; 101 Lucio Bini Mood Disorder Center, Cagliari, Italy; 102 Department of Neurosciences, Mental Health and Sensory Organs, Sant’Andrea Hospital, Sapienza University of Rome, Rome, Italy; 103 School of Medicine, Universidad Diego Portales CL, Santiago de Chile, Chile; 104 School of Pharmacy and Biomedical Sciences, University of Central Lancashire, Preston, Lancashire UK; 105 SA MRC Genomic and Precision Medicine Research Unit, Division of Human Genetics, Department of Pathology, Institute of Infectious Diseases and Molecular Medicine, University of Cape Town, Cape Town, South Africa; 106 Department of Psychiatry and Behavioral Sciences, Stanford School of Medicine, Palo Alto, CA USA; 107 Asha Bipolar Clinic, Asha Hospital, Hyderabad, Telangana India; 108 Departments of Psychiatry, Epidemiology, and Internal Medicine, Iowa Neuroscience Institute, The University of Iowa, Iowa City, IA USA; 109 Department of Neuroscience and Mental Health, Federal University of Bahia, Salvador, Brazil; 110 Bipolar Zentrum Wiener Neustadt, Sigmund Freud Privat Universität, Vienna, Austria; 111 Centre for Clinical Brain Sciences, University of Edinburgh, Edinburgh, Scotland, UK; 112 AREA, Assistance and Research in Affective Disorders, Buenos Aires, Argentina; 113 Science Directorate/Climate Science Branch, National Aeronautics and Space Administration (NASA) Langley Research Center, Hampton, VA USA; 114 Department of Psychiatry, MRC Unit On Risk & Resilience in Mental Disorders, University of Cape Town, Cape Town, South Africa; 115 College of Medicine, China Medical University (CMU), Taichung, Taiwan; 116 An-Nan Hospital, China Medical University, Tainan, Taiwan; 117 Research Division, Institute of Mental Health, Singapore, Singapore; 118 Department of Psychological Medicine, Faculty of Medicine, University of Malaya, Kuala Lumpur, Malaysia; 119 Department of Social Services and Health Care, Psychiatry, City of Helsinki, Helsinki, Finland; 120 Department of Psychiatry, Faculty of Medicine, Hasanuddin University, Makassar, Indonesia; 121 McLean Hospital, Harvard Medical School, Boston, MA USA; 122 Mood Disorder Lucio Bini Centers, Cagliari e Rome, Italy; 123 Bipolar and Depressive Disorders Unit, Hospital Clinic de Barcelona, Fundació de Recerca Clínic Barcelona-Institut d´Investigacions Biomèdiques August Pi i Sunyer (IDIBAPS), Barcelona, Spain; 124 Bipolar and Depressive Disorders UnitInstitute of Neurosciences (UBNeuro)Centro de Investigación Biomédica en Red de Salud Mental (CIBERSAM), Hospital Clinic de Barcelona, Barcelona, Spain; 125 Melbourne Neuropsychiatry Centre, Department of Psychiatry, The University of Melbourne, Melbourne, Australia; 126 Subdirección de Investigaciones Clínicas, Instituto Nacional de Psiquiatría Ramón de la Fuente Muñíz, Mexico City, Mexico; 127 Department of Psychological Medicine, Institute of Psychiatry, Psychology and Neuroscience, King’s College London, London, UK

**Keywords:** Solar insolation, Bipolar disorder, Suicide attempt, Environment, Sunlight

## Abstract

**Background:**

The rate of suicide attempts by patients with bipolar disorder is high. In addition to patient and country specific factors, environmental factors may contribute to suicidal behavior. Sunlight has multiple diverse impacts on human physiology and behavior. Solar insolation is defined as the electromagnetic energy from the sun striking a surface area on earth. We previously found that a large change in solar insolation between the minimum and maximum monthly values was associated with an increased risk of suicide attempts in patients with bipolar I disorder.

**Methods:**

The association between solar insolation and a history of suicide attempts in bipolar disorder was again investigated using an international database with 15% more data and more sites at diverse locations and countries.

**Results:**

Data were available from 5641 patients with bipolar I disorder living at a wide range of latitudes in 41 countries in both hemispheres. A large change in solar insolation between the minimum and maximum monthly values was associated with a history of suicide attempts in patients with bipolar I disorder, a replication of our prior analysis. The estimated model also associated state sponsored religion in the onset country, female gender, a history of alcohol or substance abuse, and being part of a younger birth cohort with a history of suicide attempts.

**Conclusions:**

A large change between the minimum and maximum monthly values of solar insolation was associated with a history of suicide attempts in bipolar I disorder, replicating our prior research. Physicians should be aware that daylight has wide ranging physiological and psychiatric impacts, and that living with large changes in solar insolation may be associated with an increased suicide risk.

**Supplementary Information:**

The online version contains supplementary material available at 10.1186/s40345-024-00364-5.

## Introduction

Patients with bipolar disorder display rates of suicide attempts between 31 and 35% for both bipolar types I and II (Novick 2010; Dong 2020; Tondo 2016). Diverse patient and country factors have been associated with suicide attempts in bipolar disorder. Patient factors associated with suicide attempts include female gender, depression, comorbid anxiety, alcohol or substance abuse disorder, history of trauma, comorbid personality disorder, young age of onset, rapid cycling, prior suicide attempts, family history of suicide, early life adversity, living with disabilities, and potential genetic risk factors (Schaffer 2015; MacKinnon 2005; Tondo 2016; Gonda 2012; Monson 2021; Isometsä 2014; CDC 2023). Country specific factors associated with increased suicide rates in Europe and the US include unemployment rates and economic recession (Nordt 2015; Reeves 2012). In the US, people living in rural areas have higher rates of suicide than those living in urban areas (CDC 2023). In low and middle income countries, pesticide self-poisoning remains a common method of suicide (Eddleston 2020; Karunarathne 2020).

In addition, environmental factors may also contribute to suicidal behavior in patients with bipolar disorder. The sun provides light and heat to the earth, as solar radiation warms the atmosphere and the earth’s surface. Life on earth evolved under sunlight and is entrained to the 24-h light/dark cycle. Circadian clocks are present in almost every cell of the body, and synchronization between the cells and organ systems, as well as synchronization with the solar day is required for health (Turek 2016; LeGates 2014). Sunlight widely influences human physiology, behavior, alertness, well-being, and sleep (Munch 2017). Circadian rhythm disruption is associated with disturbances in emotional responses, sleep–wake cycles, cognition, physiology, and health (Foster 2020). Virtually every psychiatric disorder is accompanied by sleep and circadian disturbances including bipolar disorder (Baglioni 2016; Meyer 2024; Leng 2019; Walker 2020). Routine treatment of patients with bipolar disorder requires ongoing risk assessment for suicidal behavior and understanding of environmental factors, including sunlight. The purpose of this study was to evaluate the association between sunlight and the history of suicide attempt in a large global sample. Based on our prior research, a large change between the minimum and maximum monthly values of solar insolation was expected to be associated with a history of suicide attempts in patients with bipolar I disorder (Bauer 2019, 2021).

## Methods

### Patient data

Researchers at university medical centers and specialty clinics, and individual practitioners collected the data by direct questioning, record review or both. Study approval was obtained from local institutional review boards according to local requirements. All patients had a diagnosis of bipolar disorder from a psychiatrist according to DSM-IV, DSM-5 or ICD criteria. The data collected for each patient included gender, age of onset, date of birth, polarity of first episode, family history of mood disorders, history of psychosis, episode course, history of alcohol or substance abuse, and history of suicide attempts. Three locations were also collected for each patient: birth location, location of onset of bipolar disorder, and current location.

### Data collection sites

There were 75 data collection sites located in 41 countries in both hemispheres. In the northern hemisphere, data collection sites were in Austria: Graz, Wiener Neustadt; Canada: Calgary, Halifax, Ottawa; China: Hong Kong; Colombia: Medellín; Denmark: Aalborg, Aarhus, Copenhagen; Ethiopia: Barhir Dar; Estonia: Tartu; Finland: Helsinki; France: Paris (2 sites);Germany: Dresden, Frankfurt, Würzburg; Greece: Athens, Thessaloniki (2 sites); India: Bengaluru, Hyderabad, Wardha; Ireland: Dublin; Israel: Beer Sheva; Italy: Cagliari, Sardinia (2 sites), Milan, Piacenza, Rome, Siena; Japan: Tokyo (3 sites); Malaysia: Kuala Lumpur; Mexico: Mexico City; Netherlands: Groningen; Norway: Oslo, Trondheim; Poland: Poznan; Russia: Khanti-Mansiysk; Serbia: Belgrade; Singapore; South Korea: Jincheon; Spain: Barcelona, Vitoria; Sweden: Gothenburg, Stockholm; Taiwan: Taichung; Thailand: Bangkok; Turkey: Ankara, Konya; Tunisia: Tunis; Uganda: Kampala; UK: Glasgow; and USA: Grand Rapids, MI, Iowa City, IA, Kansas City, KS, Los Angeles, CA, Palo Alto, CA, Rochester, MN, San Diego, CA, and Worcester, MA. In the southern hemisphere, data collection sites were in Australia: Adelaide, Melbourne/Geelong; Argentina: Buenos Aires; Brazil: Porto Alegre, Salvador, São Paulo; Chile: Santiago (2 sites); Indonesia: Mataram; New Zealand: Christchurch; and South Africa: Cape Town.

### Country data

Socioeconomic data were obtained for every country with an onset location. Data included physician density per 1000 population, country median age, unemployment rate, poverty rate, gross domestic product per capita (CIA 2024), psychiatrists per 100,000 (WHO 2019), Gini index of income inequality, percent Internet users (World Bank 2024a; 2024b), gender inequality index (UN 2024), and if the country has a state-sponsored or officially favored religion (Pew Research 2017).

### Solar insolation data

The National Aeronautics and Space Administration (NASA) POWER database provides average monthly solar insolation at a spatial resolution of 1° × 1° latitude/longitude based on 20-years of satellite observations collected from January 2001–December 2020 (NASA 2024). All solar insolation data were obtained using the NASA POWER VERSION: v9. Solar insolation measures the electromagnetic energy from the sun received for a given surface area on earth at a given time, expressed in kWh/m2/day (kilowatt hours/square meter/day).

The intensity of solar insolation is not evenly distributed across the earth’s surface but varies with the annual changes in the earth-sun orientation. Solar insolation values are affected by factors such as the angle at which the sun’s rays strike the earth’s surface, time of day, latitude, season, atmospheric conditions, and distance. The monthly pattern of solar insolation varies by latitude, with few changes throughout the year at the equator, and large changes close to the north and south poles. Tropical locations at less than 23.5° north or south of the equator may have a wet season where clouds decrease solar insolation and a dry season with clear skies rather than a winter/summer pattern. The ratio of minimum mean solar insolation/maximum mean solar insolation was calculated to reflect these changes. Additionally, locations at the same latitude but different longitude may have different solar insolation values due to local conditions such as cloud cover, altitude, atmospheric aerosols and local pollution, and proximity to large bodies of water.

To obtain solar insolation data for each patient, the actual onset locations were grouped to create reference onset locations which include all the actual onset locations within a 1 × 1 degree grid of latitude and longitude. The reference onset locations were used to obtain all solar insolation values. Additionally, solar insolation data from the southern hemisphere was shifted by 6 months for comparison to data from the northern hemisphere.

### Statistics

A significance level of 0.01 was used for all evaluations to reduce the chance of type I error. The multivariate model estimates were compared using the corrected quasi-likelihood independence model criterion (Pan 2001). Based on the logit link function, the exponentiated coefficient can be interpreted as the effect size (Li et al. 2019). Demographic variables were reported using descriptive statistics. SPSS version 29.0.02 was used for all analyses.

The generalized estimating equations (GEE) statistical technique was selected to accommodate the correlated data within reference onset locations and unbalanced number of patients between reference onset locations. The GEE technique estimates the dependent variable as a function of the entire population, producing a population averaged or marginal estimates of model coefficients (Zeger and Liang 1986). All GEE models were estimated using a binomial distribution, an exchangeable working correlation matrix and a logit link function where the patient history of suicide attempts was the dependent binary variable. An exchangeable correlation matrix was selected, to efficiently estimate GEE models with a large number of clusters including many with a single observation (Stedman 2008).

Two models were used to analyze the data. The first model repeated our prior studies (Bauer 2019; Bauer 2021). The second model modified two variables to reflect the current database. Countries hostile to religion were separated from countries with no preferred religion. A new birth cohort group was added for those who were born in 1980 or later to balance the age grouping. Four birth cohort groups were used: date of birth < 1940, ≥ 1940 and < 1960, ≥ 1960 and < 1980, and >  = 1980.

## Results

### Data

Patient data were available for 8657 patients with bipolar I disorder. Of these, data for all variables included in the best models were available for 5641 patients with bipolar I disorder. Patients with one or more missing variables were excluded from the GEE analysis. The demographics of the 5641 patients with bipolar I disorder are shown in Table 1. Supplemental Table A provides the demographic characteristics of all 8657 patients compared to the 5641 patients included in the GEE analysis.

**Table 1:** Demographics of Bipolar I patients (N = 5641)

Parameter	Value	N	%
Gender			
	Female	3204	56.8
	Male	2437	43.2
First Episode^1^			
	Manic/Hypomanic	2641	48.5
	Depressed	2800	51.5
Family History of Mood Disorder^1^			
	No	2408	46.4
	Yes	2780	53.6
Alcohol or Substance Abuse^1^			
	No	3908	69.3
	Yes	1733	30.7
State Sponsored Religion in Country of Onset^1^			
	No	2869	50.9
	Yes	2581	45.8
	Hostile	191	3.4
History of Suicide Attempts			
	No	3929	68.7
	Yes	1712	30.3
Cohort Group			
	DOB < 1940	183	3.2
	DOB > = 1940 and DOB < 1960	1334	23.6
	DOB > = 1960 and DOB < 1980	2560	45.4
	DOB > = 1980	1564	27.7
Parameter		Mean	SD
Age at time of Data Collection		47.3	14.6
Age of Onset		25.7	10.7

^1^Missing values excluded

### Onset locations

The onset locations for the 5641 patients with bipolar I disorder were in 66 countries. Of the 5641 patients, for 4695 patients (83.2%) the current city is the same as the onset city, and for 5504 patients (97.6%) the current country is the same as the onset country. Example cities with the ratio of minimum mean solar insolation/maximum mean solar insolation at varied latitudes are shown in Table 2. Of the 5641 patients 883 (15.7%) had an onset location in the tropics (less than 23.5 degrees north or south of the equator). The collection site was used as the onset location for some or all patients from Barcelona, Cape Town, Christchurch, Frankfurt, Helsinki, Melbourne/Geelong, Porto Alegro, São Paulo, Salvador, Vitoria, and Würzburg when the actual onset location was not available (Bauer 2019, 2021).

**Table 2:** Mean Ratio of Monthly Mean Minimum/Monthly Mean Maximum Insolation by Latitude for Patient Onset Locations (N = 5641)

Degrees Latitude North + South	Example reference sites	N	%	Mean ratio of monthly mean minimum/monthly mean maximum insolation
0–9		283	5.0	0.8117
	Quito, Ecuador			
	Singapore, Singapore			
	Bogota, Columbia			
	Kampala, Uganda			
	Lagos, Nigeria			
10–19		541	9.6	0.7060
	Caracas, Venezuela			
	Bangalore, India			
	Mexico City, Mexico			
	Hyderabad, India			
	Lima, Peru			
20–29		275	4.9	0.5816
	Maui, HI, US			
	Taipei, Tiawan			
	Calcutta, India			
	Sao Paulo, Brazil			
	Miami, FL, US			
30–39		1591	28.2	0.3054
	Cagliari, Italy			
	Perth, Australia			
	Valparaiso, Chile			
	Buenos Aries, Argentina			
	Capetown, South Africa			
	Tokyo, Japan			
40–49		2085	37.0	0.2021
	Halifax, Canada			
	Milano, Italy			
	Madrid, Spain			
	New York, NY, US			
	Istanbul, Turkey			
	Grand Rapids, MI, US			
50–59		625	11.1	0.0739
	Calgary, Canada			
	Poznan, Poland			
	Berlin, Germany			
	Groningen, Netherlands			
	Oslo, Norway			
	Gothenburg, Sweden			
60 +		241	4.3	0.0233
	Lillehammer, Norway			
	Trondheim, Norway			
	Helsinki, Finland			
	Nuuk, Greenland			
Total		5641	100.0	0.3068

### Model results

The best model from our prior research for explaining the association of solar insolation with a history of suicide attempts for patients with bipolar I disorder was replicated with similar results as shown in Table 3 (Bauer 2019, 2021). The parameters included in the replication are the ratio of minimum mean monthly solar insolation/maximum mean monthly solar insolation, state sponsored religion, gender, history of alcohol or substance abuse, and birth cohort.

**Table 3:** Estimated parameters for 2020 model explaining a history of suicide attempts for patients with bipolar I disorder (N = 5641)^1^

Parameters	Coefficient estimate (β)	Standard Error	Exp (β)	99% Confidence Interval		Coefficient Significance	
				Lower	Upper	Wald Chi-squared	P
Intercept	−1.036	0.2264	0.355	−1.619	−0.453	20.953	< 0.001
Ratio minimum insolation/maximum insolation	−0.655	0.1766	0.520	−1.110	−0.200	13.748	< 0.001
State sponsored religion in onset country	−0.346	0.1105	0.708	−0.631	−0.061	9.807	0.002
Male	−0.624	0.0724	0.536	−0.811	−0.438	74.335	< 0.001
History of alcohol or substance abuse	0.476	0.0703	1.610	0.295	0.657	45.974	< 0.001
DOB ≥ 1960	0.821	0.2298	2.273	0.229	1.413	12.771	< 0.001^2^
DOB ≥ 1940 and DOB < 1960	0.671	0.2100	1.955	0.130	1.212	10.198	0.001^2^

^1^GEE model using a binomial distribution and logit link function. Dependent variable: History of suicide attempts (yes/no). Model parameters: intercept, ratio of minimum insolation/maximum insolation at onset location, state sponsored religion in onset country (yes/no), alcohol or substance abuse (yes/no) and birth cohort group (DOB < 1940, DOB ≥ 1940 and DOB < 1960, DOB ≥ 1960)

^2^Individual parameters Wald chi-square statistics and significance. The model effects Wald chi-square and significance for the cohort parameter was 12.932 and 0.002 respectively with 2 degrees of freedom

Additionally, the results of the second model that includes the two modified variables, shown in Table 4, are similar to the model results in Table 3. Using the exponentiated value of the estimated coefficient as the odds ratio for a parameter (Exp (β)), the estimated coefficients for the modified model in Table 4 suggest that for every 0.1 decrease in the ratio of the minimum insolation/maximum insolation, there is a 4.5% increase in the odds of a suicide attempt. Comparing the ratio of the minimum insolation/maximum insolation of 1 near the equator and a ratio of the minimum insolation/maximum insolation of 0 at a pole, the odds of a suicide attempt increase by 45%. The estimated coefficients for the modified model suggest that being male decreases the odds of a suicide attempt by 54% and a history of alcohol or substance abuse increases the odds of a suicide attempt by 61%. The exponentiated values of the estimated coefficients for the modified model also suggest that patients born in 1980 and after increases their odds of a suicide attempt by 90% and patients born between 1960 and 1979 increases their odds of a suicide attempt by 138%.

**Table 4:** Estimated parameters explaining a history of suicide attempts for patients with bipolar I disorder (N = 5641)^1^

				99% Confidence Interval		Coefficient Significance	
Parameters	Coefficient estimate (β)	Standard Error	Exp (β)	Lower	Upper	Wald Chi-squared	P
Intercept	−0.997	0.2212	0.369	−1.567	−0.428	20.332	< 0.001
Ratio minimum insolation/maximum insolation	−0.797	0.2415	0.451	−1.419	−0.175	10.899	< 0.001
State sponsored religion in onset country−Yes	−0.369	0.1129	0.691	−0.660	−0.078	10.679	0.001^2^
State sponsored religion in onset country−Hostile	−0.623	0.2263	0.537	−1.205	−0.040	7.573	0.006^2^
Male	−0.618	0.0719	0.539	−0.803	−0.433	73.853	< 0.001
History of alcohol or substance abuse	0.478	0.0703	1.614	0.297	0.660	46.289	< 0.001
DOB ≥ 1980	0.640	0.2372	1.897	0.029	1.251	7.285	0.007
DOB ≥ 1960 and DOB < 1980	0.868	0.2295	2.381	0.276	1.459	14.290	< 0.001^2^
DOB ≥ 1940 and DOB < 1960	0.658	0.2085	1.931	0.121	1.195	9.963	0.002^2^

^1^GEE model using a binomial distribution and logit link function. Dependent variable: History of suicide attempts (yes/no). Model parameters: intercept, ratio of minimum insolation/maximum insolation at onset location, state sponsored religion in onset country (yes/no/hostile), alcohol or substance abuse (yes/no) and birth cohort group (DOB < 1940, DOB ≥ 1940 and DOB < 1960, DOB ≥ 1960 and DOB < 1980, DOB >  = 1980)

^2^Individual parameters Wald chi-square statistics and significance. The model effects Wald chi-square and significance for the state sponsored religion in onset country parameter was 14.491 and < 0.001 respectively with 2 degrees of freedom. The model effects Wald chi-square and significance for the cohort parameter was 20.256 and < 0.001 respectively with 3 degrees of freedom

## Discussion

Death by suicide remains a major public health problem and is especially of concern for patients with bipolar disorder. For patients with bipolar I disorder, a large change in solar insolation between the minimum and maximum monthly values was associated with an increased risk of a suicide attempt. A state sponsored religion in the onset country, female gender, a history of alcohol or substance abuse, and being part of a younger birth cohort were also associated with a history of suicide attempts. This international study replicated the model results from our prior research with data from 765 more patients with bipolar I disorder (5641 versus 4876 patients or 15.6% larger) (Bauer 2019, 2021). Additionally, a new model, which includes variables that better represent the current sample, provided very similar results. Multiple, and large-scale replications with heterogeneous study populations is an important approach to confirming psychological findings (Shrout 2018; McShane 2019).

Sunlight has many important, diverse and complex impacts on human physiology and behavior. Human physiology has adapted to the 24-h light dark cycle due to one rotation of the earth on its axis, displaying daily fluctuations in activity and rest (Richards 2013). Circadian timing cycles are found in nearly all human physiological processes, with clock components to produce circadian rhythms found in virtually all cells (Allada 2021; Rosenwasser 2015). However, endogenous clocks do not run at exactly 24 h, allowing adaptation to seasonal and environmental changes, and must be regularly synchronized (Duffy 2009; LeGates 2014). The primary signal that entrains the human circadian system is sunlight. Retinal ganglion cells containing the pigment melanopsin detect environmental brightness and mediate non-imaging forming visual functions including light entrainment (Benarroch 2011; Hatori 2010; Mure 2021; Hattar 2002). The suprachiasmatic nucleus (SCN) in the hypothalamus is the master circadian pacemaker over a system that includes circadian clocks genes expressed in the SCN and the rest of the brain, and almost all peripheral tissues (Silver 2014; Rosenwasser 2015). System-wide internal circadian synchronization, as well as circadian synchronization with the environment, are fundamental for good physical and mental health. Problems with the circadian system are associated with a wide range of disease states including seasonal affective disorders, optic neuropathies, migraine, sleep disturbances, neurogenerative disease, and glaucoma (Benarroch 2011; Ksendzovsky 2017; Walker 2020). Light also effects sleep via retinal ganglion cells in a circuit independent of circadian photoentrainment (Zhang 2021).

Sunlight has additional important and widespread impacts on human physiology. For example, upon exposure to sunlight, ultraviolet B radiation (UVB; 290–315 nm) is absorbed in the skin triggering vitamin D synthesis, which maintains calcium and phosphorous levels in a narrow physiological range necessary for bone development and maintenance (Holick 2024; 2016). Exposure to sunlight is the major source of vitamin D for most children and adults (Holick 2016). Optimal vitamin D levels are also required for proper functioning of the developing and adult brain (Groves 2014; Mayne 2019; Eyles 2021; Menendez 2024). Vitamin D deficiency has repeatedly been associated with an increased risk of Alzheimer’s disease and other dementia (Littlejohns 2014; Balion 2012; Chai 2019). Beyond vitamin D production, UV radiation induces local immunologic and hormonal changes that may have profound impacts on the brain and systemic body homoeostasis, and these mechanisms are being explored (Slominski 2024; 2018). Other non-visual effects of light may influence mood and learning suggesting potential negative impacts of inappropriate light exposure, such as at night (Fernandez 2018). Additionally, sensible sun exposure may reduce the risk of many chronic illness including osteoporosis, common cancers and autoimmune disorders (Holick 2024; van der Rhee 2016). Locations near the poles have the largest change in solar insolation between winter and summer, which may be a contributing factor to high suicide rates in circumpolar regions (Young 2015; Pollock 2021).

The other parameters included in the best model are consistent with prior research on suicide attempts. Alcohol and substance abuse (Sublette 2009; Østergaard 2017), and being female (Tondo 2016; Hu 2023) were significantly associated with suicide attempts. Major religions were found to be protective against suicide attempts (Lawrence 2016; Chen 2020; Rasic 2009; VanderWeele 2016). International studies have reported that suicide attempts occurred more often at a younger age (Chen 2021; Dome 2019; Simon 2007; Olfson 2017; Twenge 2018).

### Limitations

There are limitations associated with this international database. The data collection methods were not standardized, including the definition of a suicide attempt. The patient data collected did not include some variables associated with suicide such as genetic information or sexual orientation (Turecki 2016). There was no data on individual treatments taken for bipolar disorder, including the use of lithium which may decrease suicide risk. There was no data on patient general medical health, chronic diseases, sleep disturbances, serum vitamin D levels, and other medications taken which could be confounders (Ilzarbe 2023). There was no information on individual work habits or life styles that may impact sunlight exposure. The influence of online social media sites on individual suicide related behavior was not known (Luxton 2012). There was no data on the phase of bipolar disorder or season when a suicide attempt occurred, although some patients experience seasonal variation in bipolar symptoms (Geoffroy 2014). For example, a summer peak in hospital admissions for mania was reported in Finland and Denmark (Törmälehto 2022; Medici 2016), and a spring/summer peak in Taiwan (Lee 2007). There was no data on suicide deaths, which are reported higher in males than females (Hedegaard 2020; Bostwick 2016; Blisker 2011; Plans 2019). The high suicide rates in the elderly (> 75 years) with bipolar disorder was not considered (Miller 2020). The impact of the COVID 19 pandemic on suicide rates was not discussed (Sher 2020). By shifting data from the southern hemisphere by 6 months, cultural related issues related to seasonality were not included. There was no mention of how the perception of climate change may trigger anxiety, depression and thoughts of suicide (Gianfredi 2024).

Significant individual differences in the non-visual responses to light were not discussed (Spitschan 2022; Chellappa 2021; Phillips 2019). Retinal abnormalities including thinning associated with bipolar disorder were not included (Lizano 2020; Silverstein 2020). White light emitting diodes (LEDs), which have a dominant spectral wavelength in the blue light range near the peak sensitivity for the melanopsin system were not mentioned (Bauer 2018). Dangers from excessive sunlight exposure were not reviewed including risks of skin cancer and aggravation of some skin and eye diseases (Hoel 2016; van der Rhee 2016). Indoor lighting and the fundamental differences between sunlight and electric lighting were not discussed (Knoop 2020). There was no data on local temperature increases or air pollution which may be associated with suicide attempts (Aguglia 2021; Villeneuve 2023; Wu 2024; Heo 2021; Kim 2019; Radua 2024). Some patients with bipolar disorder are especially sensitive to climate and weather changes, which may be associated with suicide attempts (Di Nicola 2020). Regional variance in solar insolation occurring over decadal time frames, including dimming and brightening related to clouds and aerosols was not considered (Wild 2012; He 2018). Finally, this analysis measures association and cannot determine causality (Vieta 2024).

## Conclusions

Using an international sample, this analysis confirmed that a large change in solar insolation between the minimum and maximum monthly values was associated with an increased risk of suicide attempts in patients with bipolar I disorder. The increased risk appeared in both a replication model, and a model including modified variables with additional data. Physicians should be aware of this association, especially those who practice in in locations with a large change in solar insolation across the year. Further understanding of short and long term effects of sunlight exposure on patients with bipolar disorder is needed.

## Supplementary Information

Supplementary material 1.

## Data Availability

No datasets were generated or analysed during the current study.
