# Supplementary material for: Association between a large change between the minimum and maximum monthly values of solar insolation and a history of suicide attempts in bipolar I disorder
Source: Int J Bipolar Disord. 2024 Dec 23;12:43. doi: 10.1186/s40345-024-00364-5 (PMC11666876; doi:10.1186/s40345-024-00364-5)
Supplement: Supplementary file 1 — Supplementary material 1. [file 40345_2024_364_MOESM1_ESM.docx]

Supplemental Table A. Demographics of All Bipolar I patients (N=8657)

| Parameter | Value | | | N | % All | % Valid | % Study^1^ |
| --- | --- | --- | --- | --- | --- | --- | --- |
| Gender |  | | |  |  |  |  |
|  | Female | | | 5013 | 57.9 | 58.1 | 56.8 |
|  | Male | | | 3616 | 41.8 | 41.9 | 43.2 |
|  | Missing | | | 28 | 0.3 |  |  |
| First Episode |  | | |  |  |  |  |
|  | Manic/Hypomanic | | | 4043 | 46.7 | 49.9 | 48.5 |
|  | Depressed | | | 4064 | 46.9 | 50.1 | 51.5 |
|  | Missing | | | 550 | 6.4 |  |  |
| Family History of Mood Disorder | | | |  |  |  |  |
|  | No | | | 3614 | 41.7 | 48.0 | 45.4 |
|  | Yes | | | 3920 | 45.3 | 52.0 | 53.6 |
|  | Missing | | | 1123 | 13.0 |  |  |
| Alcohol or Substance Abuse | | | |  |  |  |  |
|  | No | | | 4238 | 49.0 | 69.0 | 69.3 |
|  | Yes | | | 1906 | 22.0 | 31.0 | 30.7 |
|  | Missing | | | 2513 | 29.0 |  |  |
| State Sponsored Religion in Country of Onset | | | |  |  |  |  |
|  | No | | | 4619 | 53.4 | 54.7 | 50.9 |
|  | Yes | | | 3511 | 40.6 | 42.6 | 45.8 |
|  | Hostile | | | 312 | 3.6 | 3.7 | 3.4 |
|  | Missing | | | 215 | 2.5 |  |  |
| History of Suicide Attempts | | | |  |  |  |  |
|  | No | | | 4800 | 55.4 | 69.6 | 68.7 |
|  | Yes | | | 2094 | 24.2 | 30.4 | 30.3 |
|  | Missing | | | 1763 | 20.4 |  |  |
| Cohort Group |  | | |  |  |  |  |
|  | DOB < 1940 | | | 336 | 3.9 | 3.9 | 3.2 |
|  | DOB >=1940 and DOB < 1960 | | | 2144 | 24.8 | 24.8 | 23.6 |
|  | DOB >=1960 and DOB < 1980 | | | 3922 | 45.3 | 45.3 | 45.4 |
|  | DOB >= 1980 | | | 2255 | 26.0 | 26.0 | 27.0 |
|  | Missing | | | 0 | 0.0 |  |  |
| Parameter |  | N Miss | % Miss | Mean | SD | Mean^1^ | SD^1^ |
| Age at time of Data Collection | | 0 | 0.0 | 47.7 | 15.0 | 47.3 | 14.6 |
| Age of Onset | | 44 | 0.1 | 25.5 | 10.7 | 25.7 | 10.7 |

^1^ Percent or value of data in the study analysis (N=5641) as shown in Table 1.
